# EFFECTS OF NORDIC WALKING IN PEOPLE WITH RESPIRATORY DISEASES: A SYSTEMATIC REVIEW AND META-ANALYSIS

**DOI:** 10.2340/jrm.v57.43090

**Published:** 2025-09-14

**Authors:** María VILANOVA-PEREIRA, Margarita BARRAL-FERNÁNDEZ, Noé LABATA-LEZAUN, Luis LLURDA-ALMUZARA, Albert PÉREZ-BELLMUNT, Cristina JÁCOME, Ana LISTA-PAZ

**Affiliations:** 1 Faculty of Physiotherapy, University of A Coruña, Galicia; 2 ACTIUM Functional Anatomy Research Group, Sant Cugat del Vallés, Barcelona; 3 Department of Physiotherapy, Universidad de Vitoria-Gasteiz (EUNEIZ), Araba; 4 Unit of Human Anatomy and Embryology, Department of Morphological Sciences, Faculty of Medicine, Universitat Autònoma de Barcelona, Spain; 5 RISE-Health, MEDCIDS-Department of Community Medicine, Information and Health Decision Sciences, Faculty of Medicine, University of Porto, Porto, Portugal; 6 Psychosocial Intervention and Functional Rehabilitation Research Group, University of A Coruña, Galicia, Spain

**Keywords:** exercise tolerance, lung diseases, Nordic Walking, respiratory tract diseases

## Abstract

**Objective:**

To systematically review and meta-analyse the effects of Nordic Walking in patients with respiratory diseases.

**Design:**

Systematic review and meta-analysis.

**Subjects/Patients:**

People with respiratory diseases.

**Methods:**

A systematic review from 9 databases and 1 trial register was conducted. Randomized controlled trials and quasi-experimental studies involving children or adults with respiratory diseases participating in Nordic Walking were included. A qualitative synthesis was conducted. When feasible, a meta-analysis was performed.

**Results:**

Thirteen studies were included, involving 514 participants. The qualitative synthesis suggested that Nordic Walking has benefits in exercise tolerance, physical activity, physical fitness, dyspnoea, lung function, and mood status. Meta-analysis was only possible for exercise tolerance, through a 6-minute walking test assessed in 7 studies, which indicated that Nordic Walking had similar effect to other interventions (mean difference 4.4; 95% confidence interval –88.1–96.9 m, *p* = 0.93).

**Conclusion:**

This systematic review demonstrates potential benefits of Nordic Walking in terms of exercise tolerance, physical activity, physical fitness, and dyspnoea, in people with respiratory diseases, comparable to other exercise forms. Further evidence is needed, particularly in studies analysing a structured Nordic Walking intervention with individually prescribed intensity.

## LAY ABSTRACT

This study looks at the benefits of Nordic Walking for people with respiratory diseases, such as asthma or chronic obstructive pulmonary disease. Nordic Walking is a type of exercise that involves walking with special poles, which can help improve physical fitness. By reviewing a large number of studies, we found that Nordic Walking could improve exercise tolerance and physical activity levels, and reduce shortness of breath in people with these conditions, comparable to other types of exercise. However, while the results are promising, more research is needed, especially studies that focus on personalized Nordic Walking programmes. This research could help healthcare providers better understand how to incorporate Nordic Walking into treatments for people with respiratory diseases, improving their quality of life.

Respiratory diseases pose a huge burden worldwide, with lower respiratory tract diseases, chronic obstructive pulmonary disease (COPD), asthma, tuberculosis, and lung cancer ranking among the 25 top conditions with highest disability-adjusted life years (DALYs) (1). Research indicates that lower levels of physical activity may contribute to an increase in DALYs, while regular exercise has been proven to be an effective intervention for managing many of these respiratory diseases (2–8).

Exercise training is a key component of pulmonary rehabilitation in patients with COPD (9). Pulmonary rehabilitation can lead to an improvement in exercise tolerance, skeletal and respiratory muscle function, efficiency of movement, symptoms of dyspnoea and fatigue, and health-related quality of life (HRQoL) (10). In patients with asthma, it has been demonstrated that exercise can reduce the use of rescue medication and exacerbations (11), and improve HRQoL, exercise tolerance (12), asthma control, and lung function (4). Exercise is part of the multidisciplinary care offered to people with cystic fibrosis and advanced lung cancer, enhancing exercise tolerance and HRQoL (5, 7). These 2 domains are also improved in prehabilitation (before surgery) and rehabilitation after lung transplantation (6). On the other hand, in acute conditions, such as COVID-19, exercise can improve dyspnoea, anxiety, muscle strength, exercise tolerance, and HRQoL (2).

It is well established that exercise has a significant dose–response relationship, with greater benefits observed at higher intensities (13). However, for patients with respiratory diseases, higher intensity exercise can pose a barrier due to increased dyspnoea and discomfort (14). In this regard, Nordic Walking (NW) is a form of exercise consisting of walking naturally, respecting the biomechanics of walking, but enhancing it by using a pair of poles for propulsion (15). This activity has been shown to be a modality of exercise that potentially generates higher values of oxygen consumption (VO_2_), heart rate (HR), and caloric expenditure, without increasing perceived exertion (16, 17). This results in greater upper body involvement compared with normal walking, engaging muscles throughout the body (18). NW has been studied in different populations demonstrating positive effects (19–21). In individuals with obesity or overweight, it has been shown to improve exercise tolerance, VO_2_, HR, and bodyweight (19–21). In cardiovascular conditions, NW has been demonstrated to be effective in improving exercise tolerance and functional fitness in people with acute coronary syndrome (22). Additionally, NW leads to improvements in exercise tolerance, physical activity, HRQoL, and grip strength in patients with heart failure (23, 24), and leads to improvements in balance, gait, and performance in activities of daily living in post-stroke survivors (25, 26). In Parkinson’s disease, NW lead to improvements in walking ability (27–29) and HRQoL (30), and also when compared with other interventions. To date, systematic reviews have been carried out in the aforementioned populations (19, 31–33) as well as in women after breast cancer (34), and in older adults (35).

There are some original studies that have been focused on the effects of NW in respiratory diseases. However, no systematic review has summarized and pooled its effects. Therefore, we aimed to systematically review and meta-analyse the effects of NW in patients with respiratory diseases.

## METHODS

### Study design

This systematic review and meta-analysis was reported following the recommendations of the Preferred Reporting Items for Systematic Reviews and Meta-Analysis (PRISMA) guidelines (36). The protocol for this review was previously registered in the International Prospective Register of Systematic Reviews (PROSPERO; CRD42022335034).

### Eligibility criteria

We included randomized controlled trials (RCT) and quasi-experimental studies involving children or adults with respiratory diseases, comparing NW (either as a standalone intervention or as part of a broader intervention) with other interventions including standard care. Studies that included patients with respiratory diseases were accepted, even if the patient cohort was mixed with individuals who had other diseases. We also considered studies without comparison arms. Studies were eligible if they reported data on at least 1 of the following outcomes: exercise tolerance, physical activity, physical fitness, dyspnoea, HRQoL, lung function, anthropometry, mood status, medication adherence, medicine intake, exacerbations, emergency department visits, hospital admissions, or work absenteeism. Feasibility outcomes such as NW adherence and adverse effects were also recorded. No language restrictions were applied; however, the text had to be suitable for accurate interpretation through translation services if the researchers were not proficient in the language.

### Information sources and search

A comprehensive search was performed in May 2024, and updated until July 2025, in 9 databases (PubMed/MEDLINE; Physiotherapy Evidence Database – PEDro; Scopus; Web of Science; Cumulative Index to Nursing and Allied Health Literature – CINAHL; Cochrane Central Register of Controlled Trials – CENTRAL; Latin American and Caribbean Health Science Information Database – LILACS; Índice Bibliográfico Español en Ciencias de la Salud – IBECS; SPORTDiscus) and in 1 register (Clinicaltrials.gov). The search focused on 2 domains (population and intervention) using a combination of standardized Medical Subject Headings (MeSH) and free-text terms, grouping with Boolean operators (AND, OR):

1. Population: respiratory tract diseases OR lung transplantation OR COVID-19 OR SARS-CoV-2 OR pulmonary diseases OR asthm* OR bronchit* OR bronchiectas* OR chronic obstructive pulmonary disease OR COPD OR lung transplantation OR lung cancer;
2. Intervention: Nordic Walking OR (walking AND Nordic) OR (walking AND poles) OR walking with poles OR pole striding OR polestriding OR nordic pole walking OR pole walking.

The participant and intervention domains were combined using the AND operator (Appendix A). The Comparison (C) domain was intentionally omitted to allow for a broader range of study designs without restricting interventions in control groups (e.g., standard care, education, other types of exercise, etc.). Furthermore, references in the included studies were screened to identify other potentially relevant studies.

### Study selection

Two authors (MVP and MBF) screened the articles independently using Covidence software (https://www.covidence.org/) (37). After removing duplicate studies, the authors initially identified relevant works by title and abstract, followed by a full-text review. In cases of disagreement, a third author (ALP) was consulted. The Cohen kappa coefficient (κ) was calculated to assess inter-rater agreement. The κ values can be interpreted as: slight agreement (≤ 0.2), fair agreement (0.21–0.4), moderate agreement (0.41–0.6), substantial agreement (0.61–0.8), and almost perfect agreement (≥ 0.81) (38).

### Data extraction

Data extraction was performed independently by 2 authors (MVP and MBF) using a standardized form that included: author’s last name and year of publication, study design, country, population, sample size (age, sex, forced expiratory volume in the first second – FEV_1_), dropouts, intervention, outcomes, and results. In cases of missing data, the authors were contacted to request the information.

### Assessment of methodological quality and risk of bias

The risk of bias and methodological quality were assessed independently by 2 authors (MVP and MBF) using the Cochrane Risk of Bias (RoB 2) tool (39) and the PEDro scale (40). The RoB 2 is used to assess the risk of bias in RCTs, and to evaluate the quality and reliability of evidence (39). The PEDro scale is specifically designed for assessing the methodological quality of RCTs from the physiotherapy field (40). In cases of disagreement, a third author (ALP) was consulted. Inter-rater reliability was calculated with κ (38).

RoB 2 assesses 5 different domains: randomization process, deviation from intended interventions, missing outcome data, measurement of the outcome, and selection of the reported result. Each domain, as well as the overall risk of bias, is classified as low (coded as green), some concerns (yellow), or high risk of bias (red) (39).

The PEDro scale assesses 11 domains, but only 10 of these are used to calculate the final score (all except the eligibility criteria). These domains include: eligibility criteria, random allocation, concealed allocation, baseline between-group similarity, blinding of participants, blinding of therapists, blinding of assessors, dropouts, intention-to-treat statistical analysis, and between-group statistical comparison, and reporting of point measures and measures of validity. Each domain is scored with 1 or 0 points depending on compliance (1 point if met). A total PEDro score lower than 4 is considered “poor”, from 4 to 5 is considered “fair”, 6 to 8 is “good”, and 9 to 10 is deemed “excellent” (40).

### Data synthesis and analysis

To analyse study and intervention characteristics, we first employed a qualitative synthesis. Results of the quantitative outcomes were qualitatively summarized. Intragroup analysis was only summarized in the case of missing intergroup analysis, or if there was no control group (CG).

Meta-analysis was performed when at least 3 studies were found using the same outcome measure. Statistical analyses were made with Review Manager 5 (Cochrane Collaboration, Oxford, UK). For continuous outcomes, sample size, post-intervention means, and standard deviations (SD) were extracted and used for the statistical analysis.

Mean difference (MD) or standard mean difference (SMD) was used as the effect size if the studies used the same or different tools/units of measurement, to measure outcomes respectively. Effect sizes were expressed with their 95% confidence interval (CI). *P* < 0.05 was considered statistically significant.

Heterogeneity was evaluated by the I^2^ statistic: I^2^ lower than 25% was considered low, between 25% and 50% moderate, and higher than 50% as high heterogeneity (41). A sensitivity analysis was performed to evaluate the consistency of the results repeating the meta-analysis but removing each article individually. Subgroups were considered by type of intervention delivered to the CG classified as no exercises (such as educational sessions or standard care) and active intervention.

## RESULTS

### Study selection

**Fig. 1:**
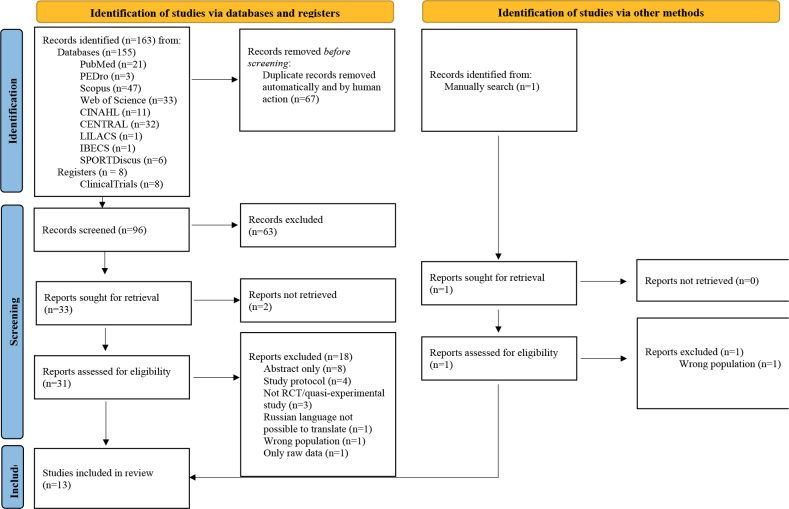
. Flow diagram of the literature search.

A search of the database yielded 155 studies (Fig. 1). After duplicates were removed, 96 titles and abstracts were screened and 63 were excluded at this stage. The remaining 33 articles were selected for full-text screening; however, 2 could not be retrieved and therefore were excluded. Twenty works were excluded, 13 met the criteria for qualitative analysis and 7 for quantitative analysis. The κ between the 2 reviewers demonstrated excellent agreement in both abstract/title (κ = 0.83, 95% CI 0.61–1.05) and full-text (κ = 0.94, 95% CI 0.59–1.29) screening.

### Study characteristics

Details regarding the included studies can be found in Table I. The 13 studies were published between 2010 and 2024. Most studies were conducted in Europe; 4 of them were conducted by the same research group in Poland (42–45). Eight were RCTs (43, 46–52) and 5 quasi-experimental studies, 4 with-out randomization (42, 44, 53, 54) and 1 without CG (45). The missing of partial data in 11 studies was due to a lack of response from authors (42–45, 49, 51, 53, 54) or because authors did not provide data (46, 47, 52).

**Table I:** Characteristics of the included studies

First author (year), country	Design	Participants	Intervention	Outcome measures	Findings
Acar et al. (2023) Turkey	RCT	Patients with COVID-19 **NWG** *n* = 15 M/F: 12/3 24 (22–49) yrs **CG** *n* = 15 M/F: 10/5 24 (22–50) yrs Dropouts: 0	**NWG** Individual Supervised in the 1st wk, unsupervised 8 wks 7 wks 3 days/wk 15 min 1st and 2nd day; 20 min 3rd day; 25 min/day 1st wk; 35 min/day 2nd wk; 45 min/day 3rd–6th wk 60-80% HR_max_ and 4–5 out of 10 (mBorg) *(Stretching, normal joint movement, and breathing exercises as warm up and cool down)* **CG** None	*Exercise tolerance*: 6MWT *Physical activity*: IPAQ-SF *Physical fitness*: Chair sit-and-stand, arm curl, two-mins step, TUG *Dyspnoea*: mMRC *Quality of life*: NHP *Mood status*: BDS	**NWG** *(pre; post; p-value intragroup, p-value intergroup)* *median (Q1-Q3)*↑6MWT (m) 448 (230-600); 580 (520–710); ***p* = 0.001; *p* = 0.001** ↑IPAQ-SF 1,139 (360–2295); 1,560 (695–3,150); *p* = 0.112; *p* = 0.967 ↑Chair sit-and-stand (rep) 14 (10–19); 18 (16–22); ***p* = 0.001; *p* = 0.041** ↑Arm curl (rep): right 21 (12–27); 25 (20–30); ***p* = 0.006; *p* = 0.018**); left (20 (10–28); 24 (20–30); ***p* = 0.001; *p* = 0.041)** ↑Two-minutes step (rep) 92 (60–130); 106 (102–168); ***p* = 0.011; *p* = 0.020** ↓TUG (s) 6 (4–9); 4 (3.5–5); ***p* = 0.001; *p* = 0.001** ↓mMRC 1 (0–3); 0 (0–2); ***p* = 0.014;** *p* = 0.380 ↓NHP: 37.40 (0–184.48); 0 (0–63.89; ***p* = 0.036;** *p* = 0.057 ↑BDS 4(0–29); 5 (0–30); *p* = 0.968; *p* = 0.631 **CG** *(pre; post; p-value intragroup) median (Q1-Q3)* ↑6MWT (m) 442.80 (200–650); 524 (470–570); *p* = 0.140 ↑IPAQ-SF 450 (150–4,050); 660 (440–1,702); *p* = 0.638 ↑Chair sit and stand (rep) 13 (10–23); 17 (13–19); *p* = 0.167 Arm curl (rep) ↓Right 23 (11–38); 20 (13–30); *p* = 0.123 ↑Left 21 (11–26); 21 (13–30); *p* = 0.505 ↓Two-minutes step (rep) 101 (71–152); 97 (90–120); ***p* = 0.020** ↓TUG (s) 6 (4–8); 5.6 (4.80–6.20); *p* = 0.051 ↓mMRC 1 (0–3); 1 (0–3); *p* = 0.157 ↓NHP 10 (0–92); 0 (0–85); *p* = 0.432 ↑BDS 2 (1–26); 5 (0–47); *p* = 0.125
Rinaldo et al. (2017) Italy	RCT	Patients with mild to severe COPD **NWG** *n* = 12 (excluding dropouts)M/F: 12/0 66.1 ± 4.5 yrs FEV_1_ 72.2 ± 18.8% pred **CG** *n* = 12 (excluding dropouts)M/F: 12/0 66.2 ± 4.2 yrs FEV_1_ 60.1 ± 24.3% pred Dropouts: 4 (2 in each group)	**NWG** Delivery mode: NR Supervised completely during first 5 wks, partially supervised during 9 wks, not supervised last 14 wks 28 wks 3 days/wk 20 min 3–4 out of 10 (mBorg) *(Extra 40 min: 10–15 repetitions of each exercise from 2–4 circuits for weight-free exercises and 3–4 out of 10 (mBorg) for aerobic classes)* **CG** Delivery mode: NR Supervised 28 wks 3 days/wk 60 min (30 min endurance, 30 min resistance) 3–4 out of 10 (mBorg) for endurance 50–80% 1RM for resistance	*Exercise tolerance*: 6MWT *Physical activity*: DEE *Physical fitness*: -Upper body and bilateral lower limb strength (1RM): leg extension and chest press -Balance: timed one-leg stance test *Quality of life*: MRF26 *Adherence*: sessions attendance	**NWG** *(pre; post; p-value post vs. pre; follow-up; p-value follow-up vs. post intragroup; p-value time x group interaction)* ↑6MWT (m) 519 ± 72; 540 ± 48; *p >* 0.05) ↓follow-up (506 ± 65; *p >* 0.05; *p* = 0.565 ↑DEE (kcal) 3,465 ± 580; 3,660 ± 974; *p >* 0.05) ↓follow-up (3,290 ± 654; *p >* 0.05; *p* = 0.852 ↑leg extension (kg) 64.1 ± 21.8; 65.6 ± 20.4; *p >* 0.05 ↓follow-up 60.2 ± 17.3; *p >* 0.05; ***p* = 0.046** ↓chest press (kg) 57.8 ± 15.1; 54.3 ± 13.9; *p >* 0.05; 49.6 ± 9.2; *p >* 0.05; ***p* = 0.011** ↑timed one-leg stance test (s) 72.5 ± 47.6; 104.6 ± 33.8; *p >* 0.05; 89.6 ± 36.3; *p >* 0.05; *p* = 0.299 ↓MRF26 5.3 ± 2.1; 2.8 ± 2.7; *p >* 0,05; 4.1 ± 2.1; *p >* 0.05; *p* = 0.096 Adherence: 100% **CG** *(pre; post; p-value post vs pre; follow-up; p-value follow-up vs post intragroup)* ↑6MWT (m) 455 ± 110; 480 ± 121; *p >* 0.05 ↓follow-up 409 ± 172; *p >* 0.05 ↑DEE (kcal) 3,070 ± 475; 3,140 ± 522; *p >* 0.05 ↓follow-up 2,890 ± 617; *p >* 0.05 ↑leg extension (kg) 59.2 ± 18.2; 65.8 ± 24.0; **p < 0.05**, ↓follow-up 54.6 ± 26.5; ***p <* 0.05** ↑chest press (kg) 41.4 ± 8.9; 50.0 ± 8.8; ***p* < 0.05**, ↓follow-up 39.5 ± 7.4 ; ***p <* 0.05** ↑timed one-leg stance test (s) 47.7 ± 48.2; 107.2 ± 22.3; *p >* 0.05; 81.4 ± 41.7; *p >* 0.05 MRF26: 7.2 ± 3.8; 4.1 ± 4.4; *p >* 0.05; 6.1 ± 4.3; *p >* 0.05 Adherence: 87%
Rutkowska et al. (2019) Poland	RCT	Patients with NSCLC at stages IIIB or IV, disqualified from surgery **NWG** *n* = 20 (excluding dropouts) M/F: 18/2 59.1 ± 6.8 yrs FEV_1_ 76 ± 16% pred **CG** *n* = 10 (excluding dropouts) M/F: 9/1 61.3 ± 8.8 yrs FEV_1_ 70 ± 23% pred Dropouts: NWG (4 chemotherapy-related events; 1 death; 1 not motivated) CG (3 chemotherapy-related event; 1 not motivated)	**NWG** Delivery mode: NR Supervised 6 wks (intervention 4 wks with 2 wks of rest in between) 5 days/wk 45 min NW NR (done or not depending on health condition and weather) *(Until complete approximately 3 h: treadmill 30–80% peak work rate* *Resistance exercise 40–70% 1RM)* **CG** None	*Exercise tolerance*: 6MWT *Physical fitness*: chair sit-and-stand, arm curl, TUG *Dyspnoea:* -mMRC -BDI -mBorg *Lung function*: spirometry (FEV_1_, FVC, FEV_1_/FVC)	**NWG** *(pre; post; p-value intragroup; p-value intergroup)* ↑6MWT (m) 486 ± 92; 531 ± 103; ***p* = 0.01;** *p* = 0.09 ↑chair sit-and-stand (rep) 13.3 ± 2.8; 14.3 ± 3.4; ***p* = 0.01;** *p* = 0.17 ↑arm curl (rep) 18.4 ± 3.1; 20.4 ± 3.5; ***p* = 0.001;** *p* = 0.36 ↓TUG (s) 6.3 ± 1.0; 6.0 ± 1.1; ***p* = 0.01; *p* = 0.01** ↑mMRC 0.7 ± 0.9; 0.7 ± 1.0; *p* = 0.18; *p* = 0.18 ↑BDI 9.5 ± 2.1; 9.5 ± 2.4; *p* = 0.83; *p* = 0.83 ↓mBorg dyspnoea scale 1.7 ± 2.2; 1.5 ± 2.1; ***p* = 0.04; *p* = 0.04** ↑FEV_1_ (% pred) 76 ± 16; 84 ± 15; ***p* = 0.01;** *p* = 0.08 ↑FVC (% pred) 87 ± 14; 95 ± 13; ***p* = 0.01;** *p* = 0.06 ↑FEV_1_/FVC (% pred) 70 ± 13; 76 ± 12; ***p* = 0.04; *p* = 0.01** **CG** *(pre; post; p-value intragroup)* ↑6MWT (m) 487 ± 100; 490 ± 124; *p* = 0.92 ↑chair sit-and-stand (rep) 11 ± 1.8; 11.2 ± 1.5; *p* = 0.34 ↑arm curl (rep) 15.2 ± 3.0; 16.2 ± 3.3; *p* = 0.06 ↑TUG (s) 6.0 ± 0.4; 6.3 ± 0.8; ***p* = 0.046** ↓mMRC 0.6 ± 1.0; 0.3 ± 0.7; *p* = 1 ↓BDI 9.9 ± 2.6; 9.8 ± 2.4; *p* = 0.72 ↑mBorg 1.1 ± 1.0; 2.6 ± 2.5; *p* = 0.42 ↓FEV_1_ (% pred) 70 ± 23; 68 ± 24; *p* = 0.68 ↑FVC (% pred) 80 ± 21; 80 ± 22; *p* = 0.83 ↓FEV_1_/FVC (% pred) 82 ± 17; 71 ± 12; ***p* = 0.03**
Jastrzebski et al. (2013) Poland	QE	Patients referred to lung transplantation (COPD, IPF, and IIP) **NWG** *n* = 22 (excluding dropouts) M/F: 22/0 50.4 yrs FEV_1_: 34% pred Dropouts: 4 (2 lung transplantation, 2 exacerbation)	**NWG** Individual Supervised 12 wks ~3 days/wk 60 min 75% HR_max_ in exercise testing, walking the distance achieved in 6MWT	*Exercise tolerance*: 6MWT *Quality of life*: SF-36 *Lung function*: spirometry (FEV_1_, FVC, FEV_1_/FVC)	**NWG** *(pre; post; p-value intragroup)* ↑6MWT (m) 310.2 ± 130.2; 372.1 ± 163.7; ***p <* 0.05** SF-36: ↑PCS 27.2 ± 8.2; 30.8 ± 7.3; ***p <* 0.05** ↓MCS 40.7 ± 11.2; 38.9 ± 8.5; *p >* 0.05 ↑FEV_1_ (% pred) 34; 42; *p >* 0.05 ↑FVC (% pred) 44; 53; ***p <* 0.05** ↑FEV_1_%VC 61; 65; *p >* 0.05
Yogeshwaran et al. (2024) India	RCT	Patients with asthma (stage I and II) **NWG** *n* = 20 FEV_1_ 1.790 ± 0.085 L **CG** *n* = 20 FEV_1_ 1.785 ± 0.081 L Dropouts: NR	**NWG** Delivery mode: NR Not supervised 4 wks 3 to 5 times/wk 30 min Intensity NR **CG** Calisthenic exercise Delivery mode: NR Not supervised 4 wks 3 to 5 times/wk 30 min/day Intensity NR	*Exercise tolerance*: 6MWT *Lung function*: spirometry (FEV_1_)	**NWG** *(pre; post; p-value intragroup)* ↑6MWT (m) 264 ± 13.92; 351.50 ± 13.09; ***p <* 0.0001** ↑FEV_1_ (L/min) 1.790 ± 0.085; 2.580 ± 0.185; ***p <* 0.0001** **CG** *(pre; post; p-value intragroup)* ↑6MWT 262.5 ± 15.52; 323 ± 15.93; ***p <* 0.0001** ↑FEV_1_ (L/min) 1.785 ± 0.081; 2.340 ± 0.105; ***p <* 0.0001**
Sivagnanam et al. (2023) India	RCT	Patients with asthma **NWG** *n* = 20 **CG** *n* = 20 Dropout: NR	**NWG** Individual Supervised 12 wks 3 days/wk 20 min Intensity: patient’s own pace *(+ 20 min: warm-up and cool-down)* **CG** Delivery mode and supervision NR 12 wks 3 days/wk 20 min bicycle ergometer training 60rpm, 75% HR_max_ *(+ 20 min: warm-up and cool-down)*	*Exercise tolerance*: 6MWT *Dyspnoea*: mBorg *Quality of life*: mAQLQ	**NWG** *(pre; post; p-value intragroup; p-value intergroup)* ↑6MWT (m) 327 ± 70.09; 616 ± 54.4; ***p <* 0.001; *p <* 0.001** ↓mBorg 7.8 ± 0.78; 5.3 ± 0.67; ***p <* 0.001; *p <* 0.001** ↑mAQLQ 30.1 ± 4.01; 59.2 ± 5.8; ***p <* 0.001; *p <* 0.001** **CG** *(pre; post; p-value intragroup)* ↑6MWT (m) 302 ± 41.0; 854.9 ± 105.9; ***p <* 0.001** ↓mBorg 7.5 ± 1.08; 2.7 ± 0.67; ***p <* 0.001** ↑mAQLQ 28.9 ± 2.37; 81.5 ± 5.4; ***p <* 0.001**
Cunningham et al. (2020) Canada	RCT (feasibility)	Patients with NSCLC, prostate, colorectal, or endometrial cancer **NWG** *n* = 4 (including dropout) M/F: 2/2 68 ± 6.4 yrs **CG** *n* = 4 M/F: 0/4 67 ± 6 yrs Dropouts: 1 (death in family)	**NWG** Individual Supervised 1 day/wk 8 wks 4 days/wk or 2 days/wk (2 participants each frequency) 20–60 min 12–15 out of 20 in Original Borg Scale **CG** None	*Exercise tolerance*: -6MWT -30-s chair stand test -UULEEX *Physical activity:* IPAQ *Physical fitness*: handgrip strength *Quality of life*: SF-36 *Anthropometry*: BMI, WC	**NWG** *(pre; post; p-value intragroup)* ↑6MWT (m) 435.9 ± 169.0; 512.3 ± 153; *p >* 0.05 ↑30 s chair stand (rep) 10.5 ± 3.7; 14.3 ± 4.2; ***p <* 0.05** ↑UULEEX (min) 10.9 ± 5.7; 11.7 ± 5.8; *p >* 0.05 ↑Handgrip strength (kg) right 25.1 ± 15.6; 30.2 ± 16.0; *p >* 0.05) left 28.1 ± 16.1; 33.0 ± 13.3; *p >* 0.05 ↑IPAQ (MET-min/week) 2346.0 ± 2532.6/5051.0 ± 3455.7; *p >* 0.05 SF-36 ↑PCS 38.60 ± 16.56; 40.45 ± 16.58; *p >* 0.05 ↑MCS 47.87 ± 15.50; 48.47 ± 20.66; *p >* 0.05 ↑BMI 23.9 ± 4.0; 25.7 ± 4.7; *p >* 0.05 ↑WC 93.0 ± 9.0; 99.2 ± 9.3; *p >* 0.05 **CG** *(pre; post; p-value intragroup)* ↑6MWT (m) 489.4 ± 77.1; 523.6 ± 46.0; *p >* 0.05 ↑30 s chair stand 9.5 ± 2.4; 11.8 ± 3.2; *p >* 0.05 ↑UULEEX (min) 13.0 ± 2.5; 13.8 ± 2.4; *p >* 0.05 Handgrip strength (kg) ↓right 23.4 ± 3.2; 23.3 ± 2.7; *p >* 0.05 ↑left 20.2 ± 4.6; 22.2 ± 5.7; *p >* 0.05 ↓IPAQ (MET-min/week) 4419.0 ± 4021.9; 1743.3 ± 1987.0; *p >* 0.05 SF-36 ↑PCS 39.74 ± 11.87; 43.96 ± 44.35; *p >* 0.05 ↓MCS 47.61 ± 13.26; 44.35 ± 12.92; *p >* 0.05 ↓BMI 29.7 ± 6.1; 29.0 ± 5.9; *p >* 0.05 ↓WC 101.1 ± 14.6; 99.5 ± 13.6; *p >* 0.05
Ochman et al. (2018) Poland	QE	Patients referred to lung transplantation (COPD, ILD) **NWG** *n* = 22 M/F: 22/0 50.4 ± 7.84 yrs FEV_1_: 39.0 ± 20.5% pred **CG** *n* = 18 M/F: 16/2 53.6 ± 8.79 yrs FEV_1_: 43.0 ± 22.2% pred Dropouts: NR	**NWG** Individual Supervised (4 out of 12 wks) 12 wks ~3 days/wk 60 min Intensity: NR **CG** None	*Exercise tolerance*: 6MWT *Dyspnoea*: -mMRC -BDI *Quality of life:* SF-36 *Lung function*: spirometry (FEV_1_, FVC, FEV_1_/FVC)	**NWG** *(pre; post; p-value intragroup; p-value intergroup)* ↑6MWT (m) 310; 373; ***p* = 0.0378**; ***p* = 0.034** -mMRC NR; NR; *p >* 0.05; ***p* = 0.002** BDI data intragroup NR; ***p <* 0.05** SF-36: ↑PCS NR; NR; ***p* = 0.011; *p* = 0.039** MCS NR FEV_1_ (% pred)intragroup NR; *p >* 0.05 ↑FVC (% pred)47.66 ± 15; 52.78; ***p* = 0.009;** *p >* 0.05 ↓FEV_1_/FVC 73.4 ± 23.6; NR; NR; *p >* 0.05 **CG** *(pre; post; p-value intragroup)* ↓6MWT (m) 326; 268; ***p* = 0.0059** ↑mMRC 3.5; NR; ***p* = 0.02** BDI data intragroup NR SF-36 data intragroup NR ↓FEV_1_ (% pred) 43.0 ± 22.2; NR; ***p* = 0.042** ↓FVC (% pred) 50.4 ± 16.5; NR; *p >* 0.05 ↑FEV_1_/FVC 81.3 ± 24.9; NR; NR
Ruban et al. (2019) Ukraine	RCT	Patients with COPD (stage IIB in remission) **NWG** *n* = 35 M/F 35/0 FEV_1_: 62.24 ± 2.37% pred **CG** *n* = 17 M/F: 17/0 FEV_1_: 63.19 ± 2.18% pred Dropouts: NR	**NWG** Group Supervised 28 days 4 times/wk 60 min 30–50 steps per min 1,500-2,500 m/day, flat terrain, inhalation 2–3 steps, exhalation 5–6 **CG** Group Supervised 28 days 4 times/wk Session duration: ~65 min General exercises for muscles (8–10 reps), massage, light tapping (40–60 beats/min), inhalers as usual	*Exercise tolerance*: 6MWT *Lung function*: spirometry (FVC, FEV_1_, FEV_1_/FVC) Stange test(s), Genchi test(s)	**NWG** *(pre; post; p-value intragroup)* ↑6MWT (m) 512.13 ± 13.10; 614 ± NR; ***p <* 0.05** ↑FVC (% pred) 63.57 ± 2.16; 72.52 ± NR; ***p <* 0.05** ↑FEV_1_ (% pred) 62.24 ± 2.37; 69.18 ± NR; *p <* 0.05 ↑FEV_1_/FVC (% pred) 65.05 ± 2.47; 72.18 ± NR; *p <* 0.05 ↑Stange test(s) 28.89 ± 2.26; NR; ***p <* 0.05** ↑Genchi test(s) 18.36 ± 1.20; NR; ***p <* 0.05** **CG** *(pre; post; p-value intragroup)* ↑6MWT (m) 532.18 ± 12.87; 555 ± NR; *p >* 0.05 ↑FVC (% pred) 64.28 ± 2.29; 66.72 ± NR; *p >* 0.05 ↑FEV_1_ (% pred) 63.19 ± 2.18; 65.12 ± NR; *p >* 0.05 ↑FEV_1_/FVC (% pred) 67.05 ± 1.29; 68.67 ± NR; *p >* 0.05 ↑Stange test(s) 32.54 ± 2.19; NR; ***p <* 0.05** ↑Genchi test(s) 21.21 ± 1.14; NR; ***p <* 0.05**
Breyer et al. (2010) Austria	QE	Patients with COPD **NWG** *n* = 30 (excluding dropouts) M/F: 14/16 61.9 ± 8.87 yrs FEV_1_: 48.1 ± 19.1% pred **CG** *n* = 30 (excluding dropouts) M/F: 13/17 59.0 ± 8.02 yrs FEV_1_: 47.1 ± 16.3% pred Dropouts = 5 (2 in NWG because of exacerbation, 3 in CG)	**NWG** Individual Supervised 3 days/wk 3 mo 60 min 75% HR_max_ **CG** None	*Exercise tolerance*: 6MWT *Physical activity*: accelerometery (McRoberts) *Quality of life*: SF-36 *Mood status*: HADS	**NWG** *(pre; post; p-value post vs. BL; 6 mo; p-value 6 mo vs BL; 12 mo; p-value 12 mo vs BL; p-value post inter-group)* ↑6MWT (m) 461 ± 154; 540 ± 159; ***p <* 0.01**; 531 ± 142; ***p <* 0.01**; 519 ± 160; ***p <* 0.01; *p <* 0.01** SF-36: ↑PCS 32.2 ± 6.50; 42.5 ± 9.62; ***p <* 0.01**; 44.1 ± 8.12; ***p <* 0.01**; 43.6 ± 9.52; ***p <* 0.01; *p <* 0.01** ↑MCS 42.8 ± 7.41; 47.2 ± 10.7; *p >* 0.05; 47.4 ± 8.91; *p >* 0.05; 46.3 ± 9.27; *p >* 0.05; NR HADS
					↓anxiety 8.8 ± 2.4; 6.6 ± 2.3; ***p <* 0.01**; 7.3 ± 2.1; ***p <* 0.01**; 7.6 ± 1.9; ***p <* 0.01; *p <* 0.01** ↓depression 9.9 ± 3.2; 6.3 ± 3.0; ***p <* 0.01**; 6.8 ± 3.0; ***p <* 0.01**; 7.9 ± 3.1; ***p <* 0.01; *p <* 0.01** **CG** *(pre; post; p-value post vs BL; 6 mo; p-value 6 mo vs BL; 12 mo; p-value 12 mo vs BL)* 6MWT (m) ↑post 436 ± 128; 442 ± 133; *p >* 0.05 ↓6 mo 428 ± 138; ***p <* 0.05** ↓12 mo 422 ± 130; ***p <* 0.01** SF-36: PCS ↑post 31.7 ± 5.79; 32.7 ± 6.39; *p >* 0.05 ↓6 mo 30.8 ± 7.40; *p >* 0.05 ↓12 mo 29.9 ± 6.89; *p >* 0.05 MCS ↑post, 6 mo 39.2 ± 9.40; 41.53 ± 12.8; *p >* 0.05; 40.7 ± 9.36; *p >* 0.05 ↓12 mo 38.7 ± 8.71; *p >* 0.05 HADS anxiety ↓post 10.5 ± 3.6; 10.2 ± 3.6; *p >* 0.05 ↑6 and 12 mo 10.5 ± 3.8; *p >* 0.05; 10.9 ± 3.6; *p >* 0.05 depression ↑post, 6 mo 11.3 ± 3.1; 11.6 ± 3.2; *p >* 0.05; 11.3 ± 3.3; *p >* 0.05 ↓12 mo 11.7 ± 3.4; *p >* 0.05 **NWG** *(variance 3 mo; p-value 3 mo vs BL; variance 6 mo; p-value 6 mo vs BL; variance 9 mo; p-value 9 mo vs BL, p-value post intergroup)* Accelerometry ↑movement intensity (m/s^2^) +0.40 ± 0.14; ***p <* 0.01**; +0.25 ± 0.09; ***p <* 0.01**; NR; NR; ***p <* 0.01** ↑time walking (min/day) +14.9 ± 1.9; ***p <* 0.01**; +12.7 ± 1.8; ***p* = 0.024**; +9.2 ± 2.9; ***p* = 0.036; *p* = 0.034** ↑time standing (min/day) +129 ± 26; ***p <* 0.01**; +133 ± 14; ***p <* 0.01**; +105 ± 4; ***p <* 0.01; *p <* 0.05** ↓time sitting (min/day) –128 ± 15; ***p <* 0.01**; -120 ± 32; ***p* = 0.016**; –101 ± 36; ***p* = 0.032; *p* = 0.014** **CG** *(variance 3 mo; p-value 3 mo vs BL; variance 6 mo; p-value 6 mo vs BL; variance 9 mo; p-value 9 mo vs BL)* Accelerometry ↓movement intensity (m/s^2^) NR; *p* = 0.385; NR; NR; NR; ***p <* 0.01** ↓time walking (min/day) NR; *p >* 0.05; NR; *p >* 0.05; NR; *p >* 0.05 ↑time standing (min/day) NR; *p >* 0.05; NR; *p >* 0.05; NR; *p >* 0.05 ↓time sitting (min/day) NR; *p >* 0.05; NR; *p >* 0.05; NR; *p >* 0.05
Kuzina et al. (2020) Rusia	QE	Patients (children) with atopic bronchial asthma **NWG:** *n* = 30 **CG**: *n* = 30 Dropout: NR	**NWG** Individual (personalized) Supervised 14 sessions Every day, every other day or twice a wk 30–90 min Between 70 and 105 steps per min **CG** Rehabilitative course: (climatotherapy, hardening, physical exercises, massage)	*Physical activity*: Physical activity level (Ruffier test) *Physical fitness*: -Abdominal muscle strength: time of holding the legs -Back muscle strength: time holding the torso *Lung function*: spirometry and peak fluorometric indices (FEV_1_, Stange test)	**NWG** *(%improvement from BL to post-intervention, p-value intragroup)* ↓Ruffier test (units) –43.0 ± 6.0; *p >* 0.05 ↑Abdominal muscle strength (s) +42.3 ± 7.0; ***p <* 0.05** ↑Back muscle strength (s) +29.4 ± 9.1; ***p <* 0.05** ↑FEV_1_ +6.6 ± 1.2; ***p <* 0.05** ↑Stange test (s) +18.8 ± 3.1; ***p <* 0.05** **CG** *(%improvement from BL to post-intervention, p-value intragroup)* ↓Ruffier test (units) –33.0 ± 6.2; *p >* 0.05 ↑Abdominal muscle strength (s) +15.1 ± 7.3; *p >* 0.05 ↑Back muscle strength (s) +13.1 ± 6.6; *p >* 0.05 ↑FEV_1_ (l) +1.1 ± 0.5; *p >* 0.05 ↑Stange test (s) +4.5 ± 2.8; *p >* 0.05
Jastrzebski et al. (2015) Poland	QE	Patients with lung cancer **NWG** *n* = 12 M/F 10/2 59 ± 7 yrs FEV_1_ 66.9 ± 13.2% pred **CG** *n* = 8 M/F NR NR FEV_1_ 67.5 ± 26.1% pred Dropout: 1 (death)	**NWG** Delivery mode supervision: NR Mean duration 7.7 wks 5 times/wk NW and aerobic, every day resistance training 45 min NW 70% of predicted HR_max_ *(+30 (aerobic) + 30 (resistance) OR respiratory muscles training and sclerometer if 6mwt < 200)* **CG** None	*Exercise tolerance*: 6MWT *Dyspnoea*: -mMRC -BDI *Quality of life*: SF-36 *Lung function*: spirometry (FEV_1_, FVC)	**NWG** *(pre; post; p-value intragroup)* ↑6MWT (m) 527.3 ± 107.4; 563.9 ± 64.6; *p* = 0.252 ↓mMRC 1.3 ± 1.1; 0.6 ± 0.5; ***p* = 0.047** ↑BDI 9.6 ± 2.3; 10.0 ± 1.5; *p* = 0.844 SF-36: ↓PCS 44.2 ± 7.6; 43.8 ± 7.3; *p* = 0.846 ↑MCS 38.0 ± 17.6; 40.3 ± 10.0; *p* = 1.000 ↑FEV_1_ (% pred) 66.9 ± 13.2; 78.4 ± 17.7; ***p* = 0.016** ↑FVC (%) 83.0 ± 16.3; 89.6 ± 22.1; *p* = 0.219 **CG** *(pre; post; p-value intragroup)* ↑6MWT (m) 502.8 ± 105; 509.4 ± 134.3; *p* = 0.816 ↑mMRC 1.4 ± 0.9; 1.8 ± 1.3; *p* = 0.313 ↑BDI 9.6 ± 2.1; 9.6 ± 3.0; *p* = 0.844 SF-36: ↓PCS 48.5 ± 8.4; 46.9 ± 10.1; *p* = 0.383 ↓MCS 49.3 ± 10.0; 48.1 ± 10.8; *p* = 0.641 ↑FEV_1_ (%) 67.5 ± 26.1; 70.3 ± 26.6; *p* = 0.844 ↑FVC (%) 79.8 ± 23.1; 81.8 ± 22.3; *p* = 0.844
Berger et al. (2018) France	RCT	Patients with moderate obstructive sleep apnoea *n* = 88 (excluding dropouts) M/F: 59/37 63 ± 7 yrs **NWG** *n* = 43 **CG** *n* = 45 Dropouts: 8 (reason NR)	**NWG** Delivery mode: NR Supervised 9 mo 3 days/wk (alternating 1 day each activity: aquagym, gymnastics, NW) 40 min Anaerobic threshold *(+10 min warm-up and + 10 min cool-down)* **CG** Two group educational sessions on healthy diet and physical activity recommendations	*Exercise tolerance*: CPET (peak VO_2_, VO_2_ at VT, O_2_ pulse) *Physical activity*: PPAQ *Anthropometry*: BMI, WC	**NWG** *(pre; change post; p-value intragroup; p-value inter-group)* ↑peak VO_2_ (ml/(min kg) 23.6 ± 6.0; +2.3 ± 3.1; ***p <* 0.001; *p* = 0.001** ↑VO_2_ at VT (ml/(min kg)) 18.2 ± 5.0; +1.8 ± 4.2; ***p <* 0.01; *p* = 0.001** PPAQ ↓Time sedentary (min/day) 497 ± 220; –11 ± 131; *p >* 0.05; *p* = 0.758 ↓LPA (min/day) 238 ± 109; –3 ± 64; *p >* 0.05; *p* = 0.667 ↑MVPA (MET/min wk) 3,295 ± 2,326; +795 ± 1,841; ***p <* 0.001; *p* = 0.002** ↑DEE (Kj/24h) 11,676 ± 2,008; +273 ± 963; ***p <* 0.01; *p* = 0.047** ↓BMI (kg/m2) 28.5 ± 4.1; -0.1 ± 0.9; *p >* 0.05; *p* = 0.422 ↑WC (cm) 100.7 ± 12.6; +0.1 ± 2.9; *p >* 0.05; *p* = 0.060 **CG** *(pre; change post; p-value intragroup)* ↑peak VO_2_ (ml/(min kg) 22.8 ± 5.7; +0.3 ± 2.4; *p >* 0.05 ↓VO_2_ at VT (ml/(min kg)) 17.4 ± 4.1; –0.4 ± 3.6; *p >* 0.05 ↑O2 pulse (ml/bpm) 12.7 ± 3.1; +0.5 ± 1.5; *p >* 0.05 PPAQ ↓Time sedentary (min/day) 489 ± 168; –2 ± 158; *p >* 0.05 ↓LPA (min/day) 261 ± 118; –4 ± 123; *p >* 0.05 ↑MVPA (MET/min week) 2,898 ± 2,087; +79 ± 1109; *p >* 0.05 ↓DEE (Kj/24h) 11556 ± 1699; –25 ± 846; *p >* 0.05 ↑BMI (kg/m2) 28.3 ± 4.3; +0.1 ± 0.9; *p >* 0.05 ↑WC (cm) 97.9 ± 12.0; +1.4 ± 3.1; ***p <* 0.01**

%: percentage; % pred: percentage predicted; 6MWT: 6-minut walking test; BDS: Beck Depression Scale; BDI: Basic Dyspnoea Index; BL: baseline; BMI: body mass index; bpm: beats per minute; CG: control group; cm: centimetres; COPD: chronic obstructive pulmonary disease; CPET: Maximal Cardiopulmonary Exercise Test; DEE: daily energy expenditure; FEV_1_: Forced expiratory volume in first second; FVC: forced vital capacity; HADS: Hospital Anxiety and Depression Scale; HR_max_: maximum heart rate; IIP: idiopathic interstitial pneumonia; ILD: interstitial lung disease; IPF: idiopathic pulmonary fibrosis; IPAQ-SF: International Physical Activity Questionnaire – Short Form; kcal: kilocalorie; kg: kilogram; L: litres; LPA: light physical activity; m: metres; mAQLQ: mini Asthma Quality of Life Questionnaire; mBorg: modified Borg Rating of Perceived Dyspnoea Scale; MCS: Mental Component Summary; MET: Metabolic Equivalent of Task; M/F: male/female ratio; min: minute; ml: millilitres; mMRC: Modified Medical Research Council; mo: months; MRF26: Maugeri Respiratory Failure Questionnaire; m/s^2^; metres per second squared; MVPA: moderate-to-vigorous physical activity; NHP: Nottingham Health Profile; NR: not reported; NSCLC: non-small cell lung cancer; NW: Nordic Walking; NWG: Nordic Walking Group; PCS: Physical Component Summary; PEF: peak expiratory flow; post: post-intervention; PPAQ: Population Physical Activity Questionnaire; pre: pre-intervention; Q1: quartile 1; Q3: quartile 3; QE: quasi-experimental study; RCT: randomized controlled trial; Rep: repetitions; RM: maximum repetition; s: seconds; SF-36: Short Form 36 Health Survey; TUG: Timed Up and Go Test; VO_2_: volume of oxygen consumption; VO_2_ at VT: volume of oxygen consumption at ventilatory threshold; UULEX: Upper Limb Exercise Test; wk: week; wks: weeks; yrs: years.

### Population

A total of 514 participants were included in the studies. Thirty participants abandoned the trials before finishing (5.86% of total), but this information was available in only 8 studies (42, 43, 45, 48–51, 54). Only 1 study focused on a paediatric population (53). For this reason, this study was excluded from the meta-analysis. In the studies conducted with adults, the median age of participants was 60.2 ± 8.8 years old. Three of them recruited only patients under 40 (51), 55 (50), or 60 years old (49).

Three studies included patients with COPD (49, 52, 54), 3 with asthma (46, 47, 53), 3 with lung cancer (42, 43, 50), and 2 included patients referred for lung transplantation (44, 45). The remaining studies involv-ed populations with obstructive sleep apnoea (OSA) (51) and COVID-19 (48). The baseline FEV_1_ ranged from 34% (45) to 76% (43).

### Intervention

Ten studies implement NW as the sole intervention in the Nordic Walking group (NWG) (44–48, 50–54), while 3 incorporated NW as part of a multi-component intervention (42,43,49). These multi-component interventions combined NW with treadmill and resistance exercises (43); aerobic and resistance training, or respiratory muscle training and sclerometer (42); weight-free circuit and aerobic exercise (49). Of the 8 studies that report the delivery mode (44, 45, 47, 48, 50, 52–54), only 1 implemented NW as a group intervention (52), while the remainder conducted the intervention individually. In 7 of the 13 studies (54%), NW was conducted under complete supervision (43, 45, 47, 51–54), while in 4 it was partially supervised (44, 48–50). One study did not supervise any session (46) and another did not report this information (47). The shortest NW intervention lasted 4 weeks (46, 52), while the longest lasted 9 months (51). The frequency of sessions per week ranged from 2 to 7 (42–54), with 3 per week being the most common (44, 45, 47–49, 51, 54). The duration of an NW session varied from 15 (48) to 90 min (53).

The methods for monitoring exercise intensity varied across studies. Four studies used the percentage of maximal heart rate (HR_max_) with the target intensities set at the range 60–80% (42, 45, 48, 54). Rating scales for perceived dyspnoea and fatigue were employed in 3 studies: the modified Borg scale, with a range from 3 to 5 (48, 49) and the original Borg scale, with a target range from 12 to 15 (50). Two studies measured intensity using steps per minute, ranging from 70 to 105, and from 30 to 50 respectively (52, 53). In one study, the intensity was determined by the patient’s own pace (47). The remaining studies did not report the intensity used (43, 44, 46).

Regarding the comparison arm, 4 studies compared NW with other forms of exercise: endurance and resistance training (49), calisthenic exercise (46), bicycle ergometer training (47), and general muscle exercises combined with massage (52). Two studies included an educational programme (51, 53) and the remaining 6 provided standard care (42–44, 48, 50, 54). Only 1 study did not have a comparison arm (45).

### Methodological quality and risk of bias

**Fig. 2:**
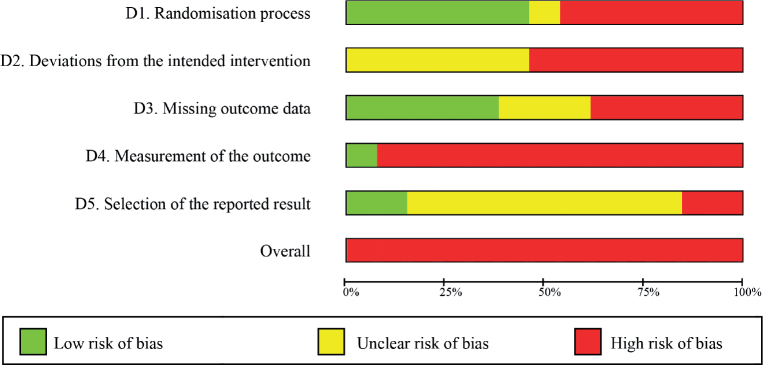
Risk of bias graph.

The result of RoB 2 tool is shown in Appendix B, and risk of bias graph in Fig. 2. All the reports had an overall “high risk” result. Two studies had a “high risk” in all domains (44, 53), and another had it in 4 domains (45). All studies but the study by Berger et al. (51) had a “high risk” in domain 4 (“Measurement of the outcome”). For the inter-rater reliability for the RoB 2 tool between the 2 independent reviewers, the κ was 0.41 (95% CI 0.2–0.63). The average score for the PEDro scale was 4.61/10, which is considered “fair” methodological quality (Table II). The κ for the PEDro scale between the reviewers was 0.7 (95% CI 0.53–0.88).

**Table II:** PEDro Scale

First author, year	Eligibility criteria	Random allocation	Concealed allocation	Baseline similarity	Blind subjects	Blind therapists	Blind assessor	Measures 85% of the sample	Intention-to-treat analysis	Between-group comparisons	Point measures and variability	Total*
Breyer, 2010	0	1	1	1	0	0	0	1	0	1	1	6
Jastrzebski, 2013	0	0	0	0	0	0	0	1	0	0	1	2
Jastrzebski, 2015	0	0	0	0	0	0	0	1	0	0	1	2
Rinaldo, 2017	1	1	1	0	0	0	0	1	0	1	1	6
Ochman, 2018	0	0	0	1	0	0	0	0	0	1	0	2
Cunningham, 2019	0	1	1	1	0	0	0	1	0	0	1	5
Ruban, 2019	0	1	0	1	0	0	0	1	1	0	1	5
Rutkowska, 2019	1	1	1	1	0	0	0	0	0	1	1	6
Acar, 2023	0	1	1	0	0	0	0	1	1	1	1	6
Kuzina, 2023	0	0	0	0	0	0	0	0	0	1	1	2
Yogeshwaran, 2024	1	1	0	0	0	0	0	0	0	1	1	4
Sigvagnanam, 2023	1	1	0	1	0	0	0	1	1	1	1	7
Yogeshwaran, 2024	1	1	0	0	0	0	0	0	0	1	1	4

### Effect of NW

**Fig. 3:**
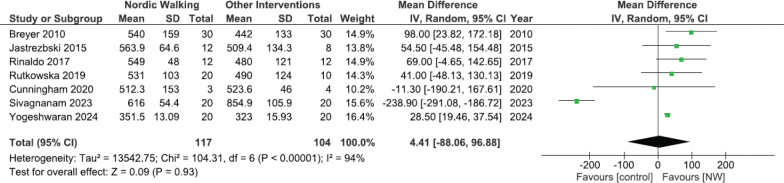
Comparison of 6 minutes walking test (6MWT) (m) between Nordic Walking (NW) and control groups (CG). CI: confidence interval; I^2^: heterogeneity statistic; IV: inverse variance.

**Fig 4:**
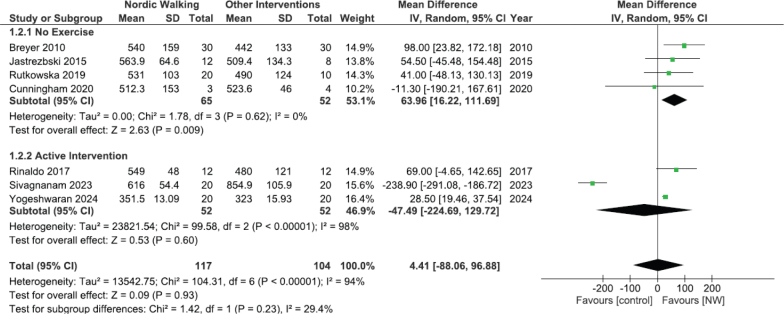
Comparison of 6 minutes walking test (6MWT) (m) between Nordic Walking (NW) and control groups (CG) differentiating between no exercise intervention and active intervention. CI: confidence interval; I^2^: heterogeneity statistic; IV: inverse variance.

*Exercise tolerance.* From the 13 included studies, the 6MWT was used to evaluate exercise tolerance in 11 studies (42–50,52,54). Meta-analysis was made for 6MWT distance (Fig. 3) based on data from 7 studies (42, 43, 46, 47, 49, 50, 54). The 4 additional studies were excluded from meta-analysis due to reporting data as median and quartiles (48), providing incomplete data (44, 52), or lacking a CG (45). The overall MD was 4.41 m (95% CI –88.06–96.88) and the overall effect Z = 0.09 (*p* = 0.93). Heterogeneity was considered high (I^2^ = 94%) (Appendix C, Table C.1.). The sensitivity analysis showed a relevant difference when excluding the study by Sivagnanam et al. (47) (Appendix C, Table C.2.) (55). Excluding this study from the meta-analysis, NWG covered a significantly greater distance than the CG in the 6MWT (MD 30.29 m (95% CI 21.47–39.1) I^2^ = 0%), difference statistically significant (*p* < 0.00001) (Appendix D, Fig. D.1). Subgroup analysis was made comparing NW with no exercise intervention (Fig. 4). The NWG achieved a statistically significant increase in 6MWT distance (MD 63.96 m; 95% CI 16.22–111.69; *p* = 0.009), and the I^2^ value suggested a very low heterogeneity (I^2^ = 0%). The comparison between NW and other active interventions had a high heterogeneity (I^2^ = 98%) and a non-significant overall effect (*p* = 0.60). Cunningham et al. (50) was the only study to measure exercise tolerance using the 30-s chair stand test and the Upper Limb Exercise Test (UULEX). Intragroup analysis shows a significant improvement in NWG only with 30-s chair stand test. Berger et al. (51) used a cardiopulmonary exercise test and revealed a statistically significant increase in the peak oxygen consumption (peak VO_2_), in VO_2_ at ventilatory threshold and in the oxygen pulse in the NWG compared with CG.

*Physical activity*. Physical activity was assessed in 6 studies (48–51, 53, 54), using 5 different tools: the International Physical Activity Questionnaire – Short Form (IPAQ-SF) (48, 50); daily energy expenditure (DEE) (49); the Population Physical Activity Questionnaire (51); accelerometry (54); and the Ruffier test (53). One study found NWG engaged in more moderate to vigorous physical activity and had a higher DEE (51). In another study, participants in NWG performed more intense movements, spent more time walking and standing, and less time sitting, compared with CG, after intervention and after follow-up (6 months later) (54). The remaining 4 studies did not show differences between groups (48–50, 53).

*Physical fitness.* The outcome was quantified in 5 studies (43, 48–50, 53), with different tools. Intergroup analysis revealed that NWG exhibited superior outcomes compared with CG only in the chair sit-and-stand test, arm curl test, and two-minute step test (48), and took fewer seconds to complete the Timed Up and Go test (43, 48). Intragroup analysis showed only an increase in abdominal and back muscle strength in NWG (53). The other studies did not reveal any significant differences between the groups (43, 48–50).

*Dyspnoea.* Dyspnoea was assessed in 5 studies (42–44, 47, 48). Four studies used the modified Medical Research Council scale (mMRC) (42–44, 48), 3 employed the Baseline Dyspnoea Index (BDI) (42–44), and 2 the modified Borg scale (43, 47). In 1 the mMRC and BDI showed better outcomes in NWG in comparison with the CG during post-intervention measurement (44). Regarding the modified Borg scale, both studies reported superior outcomes in the NWG compared with the CG in the post-intervention assessment (43, 47). Intragroup analysis showed an improvement only in NWG measured with mMRC (42, 48).

*Health-related quality of life.* This outcome was measured in 8 studies (42, 44, 45, 47–50, 54). The most frequently employed instrument was the Short Form-36 Health Survey (SF-36) (42, 44, 45, 50, 54); other tools included the Nottingham Health Profile (NHP) (48), the Maugeri Respiratory Failure Questionnaire (MRF26) (49), and the mini Asthma Quality of Life Questionnaire (mAQLQ) (47). When comparing the 2 groups, NWG exhibited superior outcomes in SF-36 post-intervention (44, 54) and at follow-up (54). Intragroup analysis shows an improvement in physical component summary (PCS) in NWG in 2 studies (45, 54). In the NHP only NWG improved significantly after intervention, but there was no difference between groups (48). In the mAQLQ, CG had better results than NWG (47).

*Lung function.* The assessment of lung function was conducted in 7 studies (42–46, 52, 53). The parameters analysed included: spirometry (42–46, 52, 53), the Stange test (52, 53), and the Genchi test (52). No significant changes were observed in any of the individual intergroup analyses. Intragroup, in 1 study both groups improved significantly FEV_1_ (46) while in the other 3 this improvement appeared only in NWG (42, 43, 53). Forced vital capacity (FVC) was improved in NWG in 3 other studies (43–45), and CG had a significant decrease in its value in 1 study (44). CG also decreased FEV_1_/FVC in 1 study (43). Ruban et al. (52) presented a significant increase in the Stange test and Genchi test in both groups, and in Kuzina et al. (53) only NWG improved in the Stange test.

*Anthropometry.* Two studies examined anthropometric variables (50,51). The parameters assessed were body mass index (BMI) and waist circumference, but no significant changes were reported.

*Mood status.* Only 2 studies assessed participants’ mood status (48, 54), using the Hospital Anxiety and Depression Scale (HADS) (54) and the Beck Depression Scale (BDS) (48). HADS revealed a significant reduction in both anxiety and depression scores in the NWG compared with the CG in post-intervention and at all follow-up measurements (54). With the BDS, no changes were seen (48).

*Adherence.* Rinaldo et al. (49) was the only study to measure sessions attendance between groups, and no significant differences were observed.

*Adverse effects.* Of the 13 studies, 6 reported not having any adverse effects (43–45, 49–51). One specified that they did not have “serious” adverse effects, but reported 2 dropouts in NWG due to exacerbations (in the CG, there were 3 dropouts, but the reasons were not reported) (54). The remaining 6 studies did not provide information on adverse effects (42, 46–48, 52, 53).

## DISCUSSION

This systematic review and meta-analysis suggest that NW may be effective in improving exercise tolerance, physical activity, physical fitness, and dyspnoea, with benefits comparable to those achieved through other forms of exercise. NW was not superior to educational sessions and standard care in terms of anthropometry and adherence. Results related to HRQoL, mood status, and lung function were inconclusive.

In terms of exercise tolerance, measured with the 6MWT, NW did not demonstrate superiority over other interventions regarding distance walking (MD 4.41 m; 95% CI –88.06–96.88) with the difference not reaching statistical or clinical significance. The minimal clinically important difference (MCID) for the 6MWT is set at 26 m for individuals with asthma (56), between 25 and 35 m for patients with COPD (57), from 22 to 42 m in patients with lung cancer (58), and from 29 to 34 m for those with idiopathic pulmonary fibrosis (59). A previous systematic review by Bohannon et al. (60) established the MCID range as 14 to 30.5 m for various respiratory patient groups. The non-significant result could be related to the substantial heterogeneity across articles (I^2^ = 94%). Sensitivity analysis identified Sivagnanam et al. (47) as an outlier, reducing the I^2^ until 0% if it was not included in analysis, which may be explained by the fact that in this study CG achieved significantly better 6MWT distance results than NWG (855 m and 618 m respectively, *p* < 0.001). One potential explanation for the CG’s superior outcomes may be the type and intensity of exercise prescribed: the CG trained on a cycle ergometer at 75% of their HR_max_, while the NWG performed NW at a self-selected pace, without specific intensity guidance (47). This lack of guidance probably led the NWG to exercise at lower intensity than the CG, even potentially below recommended levels for individuals with respiratory diseases (55). Excluding this study from the meta-analysis, the NWG covered a significantly greater distance than the CG in the 6MWT. Furthermore, the difference in 6MWT distance achieved by the NWG compared with the CG would be sufficiently large to be considered clinically meaningful. The benefits of exercise are well established, as is the dose–response relationship, with greater benefits observed as the dosage of exercise increases in terms of intensity, frequency, and duration (61).

Given the overall meta-analysis result, which show-ed a non-significant difference between interventions – probably influenced by the Sivagnanam et al. (47) study – a subgroup meta-analysis was conducted. This analysis was based on the type of CG intervention (no exercise intervention vs active intervention). When comparing NW with no exercise intervention, the NWG achieved a statistically significant increase in 6MWT distance (MD 63.96 m; 95% CI 16.22–111.69; *p* = 0.009), with no heterogeneity (I^2^ = 0%). However, when NW was compared with other active interventions, the high heterogeneity (I^2^ = 98%) and the non-significant overall effect (*p* = 0.60) prevent us from drawing conclusions regarding the relative efficacy of NW vs other exercise types. We hypothesize that NW may be superior to no exercise intervention, but does not outperform other forms of exercise (endurance, resistance, or calisthenic exercise). Additionally, in this subgroup analysis, Sivagnanam et al. (47) again appears as the dissenting article, while the remaining studies indicated that NW tends to improve 6MWT distance more than no exercise intervention (46, 49). In 1 study, exercise intensity was not reported (46), and in the other, both groups trained at the same intensity (49). We further hypothesize that for NW to yield results comparable to other forms of exercise, it must be prescribed according to general exercise recommendations (55) and individually tailored to be at least as effective as other exercise types. To determine whether NW is superior to other forms of exercise, future research should consider a 3-arm RCT comparing NW, cycle ergometer, or treadmill, and no exercise intervention, with a standardized exercise dosage (minimum of 8 weeks, 2–3 days per week, 20–60 min per session, at a minimum intensity of 60% of HR_max_) (55). In terms of exercise tolerance, other variables were used: 30-s chair stand test, CPET, and UULEEX (50, 51). Future reviews may enable the inclusion of these measures in a meta-analysis.

Given the comparable benefits of Nordic Walking and other exercise interventions, clinicians should consider patient preference and accessibility when recommending an exercise modality. Notably, NW allows patients to achieve a higher exercise intensity compared with conventional walking, without a proportional increase in perceived physical exertion or fatigue (16, 17), which may be a compelling reason to recommend NW for patients with respiratory diseases.

Physical activity levels increased following the NW intervention in 2 (51, 54) out of 6 studies that measured this outcome (48–51, 53, 54), but comparisons are difficult as distinct outcomes, both subjective and objective, were used. One study focused on patients with COPD (54), while the other involved individuals with OSA (51). These findings align with those reported by the authors of this review in terms of COPD (62). Conversely, the remaining studies did not find significant evidence that NW enhances physical activity levels. However, a review by Troosters et al. (63) suggests that, although implementing strategies to increase physical activity can be challenging, improving exercise tolerance may serve as a promising first step toward achieving this goal. In this context, NW could serve as a viable intervention option.

In terms of physical fitness, 3 studies reported significant differences with NW (43, 48, 53). Notably, these 3 studies had a CG that received only standard care. The areas of improvement included leg and arm muscle strength and endurance, balance, and gait. For individuals with respiratory diseases, maintaining or enhancing physical fitness is essential, as the disease itself (55), or related factors such as hospitalizations (64), can lead to a decline in skeletal muscle mass and function. Addressing the cycle of progressive muscle dysfunction, which leads to reduced physical activity, should be a priority (65). It is also important to note that Kuzina et al. (53) measured abdominal and back muscle strength based on the duration of holding the legs and torso, respectively. This method may not be the most appropriate for assessing strength, as it measures time, which is more closely related to endurance than to muscle strength.

Two studies reported higher HRQoL in the NWG compared with the CG following the intervention (44, 54). While the study from Sivagnanam et al. (47) observed the opposite trend, as previously discussed, its findings differed from those of the other studies. There is moderate evidence suggesting that physical activity positively impacts quality of life (66). In this domain, the determining factor may be the presence of an exercise intervention, as the CG that experienced enhanced HRQoL also participated in an exercise protocol.

Regarding lung function, anthropometry, and mood status, the current evidence remains insufficient, highlighting the need for future studies to better clarify whether any benefits exist after implementing an NW programme in patients with respiratory diseases.

Adherence was only assessed in only 1 study, which found no significant differences between groups (49). The interventions for both groups were similar in structure, differing only in the type of exercise performed, which may explain this. Of 514 total participants, only 30 dropped out of the study, which means 5.86% of the total, lower than observed in other studies (67). When comparing withdrawals between groups, in those studies that reported it and specified the reference group, the rate is similar in both groups (43, 45, 49, 50, 54). These results support our belief that NW may be well accepted and well tolerated by patients with respiratory diseases. Future research should evaluate adherence rates and document dropout rates, as well as patient experience with this training modality, to provide a more comprehensive assessment of the acceptability of NW.

This is the first systematic review focused on the effects of NW in individuals with respiratory diseases. It includes a total of 13 articles, which generally exhibit low methodological quality, mainly related with missing information related to the randomization process. These low-quality results are influenced in 4 of the studies (47–50) only by domain 4 of the RoB 2 scale, “measurement of the outcome”. A randomization method and intention-to-treat analysis should be implemented by the authors to minimize the risk of bias. Meta-analysis was only feasible for exercise tolerance due to the limited number of articles and the variability in data presentation among them. Finally, in 1 of the studies, NW was only performed if the weather conditions allowed it, but they did not specify the percentage of compliance with the sessions (43). Future trials should attempt to improve the rigour of their design and reporting.

### Limitations and strengths

This study has several limitations that should be acknowledged. One of the studies was translated from Russian with the tool “DEEPL-Pro”, resulting in an imperfect translation that made reading the article challenging (53). Missing data restricted the ability to conduct a meta-analysis on outcomes beyond the 6MWT. The authors of 12 studies were contacted to address these deficiencies (42–44, 46–54). Unfortunately, 8 of them did not respond to our enquiry (42–44, 48, 49, 51, 53, 54). Among the 4 who replied, only 1 provided the specific information requested (50). The other 3 either had lost their data (52), no longer had it stored (46), or did not answer all questions (47); however, they clarified some intervention details. Beyond these limitations regarding meta-analysis, the heterogeneity among the included clinical conditions poses an additional challenge to the interpretation of the findings. Differences in underlying pathophysiological mechanisms and disease progression trajectories may influence treatment responses and limit the generalizability of the pooled results. Third, we included 1 study involving mixed cancer types, half of which were lung cancer patients, to ensure all respiratory disease studies were covered. Although this could be seen as a limitation due to the absence of lung cancer-specific results, sensitivity analysis showed that excluding this study did not change the meta-analysis results. Moreover, sample sizes were small in most studies; 11 studies included 40 or fewer participants, and the remaining 2 had a total sample of 60 (53, 54). Fifth, only 1 study included children, so conclusions cannot be extrapolated to paediatric populations. Furthermore, only 2 studies included assessments after the follow-up period (49, 54), so no conclusions concerning the long-term effects could be drawn. There was also significant variability in the intervention across the included studies, hindering comparisons and leading to inconclusive results. To mitigate this, a meta-analysis was conducted through post-hoc subgroup analyses, as these analyses were not pre-specified in the PROSPERO registration (68). This implies that the results obtained from these analyses should be interpreted with caution but could help clarify new lines of future research. Next, the included NW interventions (NWG) were heterogeneous too, with variations in dosage and the combination of NW with other forms of exercise in 2 studies (43, 49). These inconsistencies further complicate the interpretation of the results and emphasize the need for standardized intervention protocols in future research. Lastly, the wide range of variables assessed across studies and the inconsistency in measurement instruments again hindered the ability to draw definitive conclusions, as few variables or instruments were consistently used.

Among the strengths, this is the first systematic review to focus on the effects of NW on individuals with respiratory diseases. The scope of this work is broad, encompassing studies on NW across various respiratory conditions, rather than limiting the review to a single disease. Additionally, quasi-experimental studies were included, the review not being restricted to RCTs. We did not apply any restrictions regarding the language in which the articles were written. When feasible, a meta-analysis was conducted. To enhance the depth of analysis and data interpretation, subgroup analyses were also performed.

### Conclusion

NW has demonstrated benefits for patients with respiratory diseases in terms of exercise tolerance, physical activity, physical fitness, and dyspnoea, comparable to other forms of exercise. However, no differences were observed between interventions regarding HRQoL, lung function, anthropometry, mood status, or adherence. The positive outcomes seem to be more closely linked to the general benefits of exercise rather than NW as a specific intervention. Given the heterogeneity in the data and measurement instruments, small sample sizes, and the high risk of bias across studies, the conclusions of this review should be interpreted with caution. Further research is needed to compare NW with other forms of exercise, ensuring equivalent exercise dosages.

## Supplementary Material


