# Supplementary material for: EFFECTS OF NORDIC WALKING IN PEOPLE WITH RESPIRATORY DISEASES: A SYSTEMATIC REVIEW AND META-ANALYSIS
Source: J Rehabil Med. 2025 Sep 14;57:43090. doi: 10.2340/jrm.v57.43090 (PMC12447519; doi:10.2340/jrm.v57.43090)
Supplement: Supplementary file 1 [file JRM-57-43090-s1.pdf]

Supplementary material has been published as submitted. It has not been copyedited, or typeset by Journal of Rehabilitation Medicine

Appendix A. Search strategy

1. **Search strategy for PubMed (MEDLINE)**

**PARTICIPANTS**	("Respiratory Tract Diseases" (Mesh) OR "Lung Transplantation" (Mesh) OR "COVID-19" (Mesh) OR "SARS-CoV-2" (Mesh) OR "Pulmonary diseases" (tiab) OR "asthm*" (tiab) OR "bronchit*" (tiab) OR "bronchiectas*" (tiab) OR "Chronic Obstructive Pulmonary Disease" (tiab) OR "COPD" (tiab) OR "lung transplantation" (tiab) OR "covid19" (tiab) OR "covid-19" (tiab) OR "lung cancer" (tiab))
**INTERVENTION**	("Nordic Walking" (Mesh) OR ("Walking" (Mesh) AND ("Nordic" (tiab) OR "Poles" (tiab))) OR "Nordic Walking" (tiab) OR "Walking with poles" (tiab) OR "pole striding" (tiab) OR "polestriding" (tiab) OR "nordic pole walking" (tiab) OR "pole walking" (tiab))
Note: Keyword groups will be linked by AND	

2. **Search strategy for PEDro**

**PARTICIPANTS**	"nordic walking" OR "pole walking"
**INTERVENTION**	"lung diseases" OR "respiratory diseases" OR "respiratory disorders" OR "asthma" OR "copd" OR "lung transplantation" OR "bronchitis"
Note: Each keyword of one group was linked with AND individually with one keyword of the other group, which made a total of 14 combintations	

3. **Search strategy for Scopus**

**PARTICIPANTS**	((TITLE-ABS-KEY("Respiratory Tract Diseas*") OR TITLE-ABS-KEY("Lung transplantation") OR TITLE-ABS-KEY("Pulmonary diseases") OR TITLE-ABS-KEY("lung diseas*") OR TITLE-ABS-KEY("respiratory diseas*") OR TITLE-ABS-KEY("respiration disorder*") OR TITLE-ABS-KEY("asthm*") OR TITLE-ABS-KEY("bronchit*") OR TITLE-ABS-KEY("bronchiectas*") OR TITLE-ABS-KEY("Pulmonary Disease, Chronic Obstructive") OR TITLE-ABS-KEY("Chronic Obstructive Pulmonary Disease") OR TITLE-ABS-KEY("COPD") OR TITLE-ABS-KEY("lung cancer") OR TITLE-ABS-KEY("Sleep Apnea") OR TITLE-ABS-KEY("covid-19") OR TITLE-ABS-KEY("covid19") OR TITLE-ABS-KEY("covid 19") OR TITLE-ABS-KEY("SARS-CoV-2"))
**INTERVENTION**	((TITLE-ABS-KEY("Nordic Walking") OR TITLE-ABS-KEY("Walking with poles") OR TITLE-ABS-KEY("Pole striding") OR TITLE-ABS-KEY("polestriding") OR TITLE-ABS-KEY("nordic pole walking") OR TITLE-ABS-KEY("pole walking"))
Note: Keyword groups will be linked by AND	

4. **Search strategy for Web of Science**

**PARTICIPANTS**	((TITLE-ABS-KEY("Nordic Walking") OR TITLE-ABS-KEY("Walking with poles") OR TITLE-ABS-KEY("Pole striding") OR TITLE-ABS-KEY("polestriding") OR TITLE-ABS-KEY("nordic pole walking") OR TITLE-ABS-KEY("pole walking"))
**INTERVENTION**	("respiratory tract diseas*" (Topic) or "lung transplantation" (Topic) or "pulmonary diseas*" (Topic) or "lung diseas*" (Topic) or "respiratory diseas*" (Topic) or "respiration disorder*" (Topic) or "asthm*" (Topic) or "bronchit*" (Topic) or "bronchiectas*" (Topic) or "Pulmonary Disease, Chronic Obstructive" (Topic) or "Chronic Obstructive Pulmonary Disease" (Topic) or "COPD" (Topic) or "Lung cancer" (Topic) or "sleep apnea" (Topic) or "covid 19" (Topic) or "covid-19" (Topic) or "covid19" (Topic) or "SARS-CoV-2" (Topic))
Note: Keyword groups will be linked by AND	

5. **Search strategy for CINAHL**

**PARTICIPANTS**	((MM "Respiratory Tract Diseases") OR "Respiratory tract diseas*" OR "lung transplant*" OR "pulmonary diseas*" OR "lung diseas*" OR "respiratory diseas*" OR "respiration disorder*" OR "asthm*" OR "bronchit*" OR "bronchiectas*" OR "chronic obstructive pulmonary disease" OR "COPD" OR (MM "COVID-19+") OR "covid19" OR "covid 19" OR "covid-19" OR "SARS-CoV-2" OR "Lung cancer" OR "SARS-CoV-2")
**INTERVENTION**	(MM "Nordic Walking") OR "nordic walking" OR "walking with poles" OR "pole striding" OR "Polestriding" OR "Nordic Pole Walking" OR "pole walking")
Note: Keyword groups will be linked by AND	

6. **Search strategy for Cochrane CENTRAL**

**PARTICIPANTS**	((MeSH descriptor: (Lung Transplantation) explode all tres) OR (MeSH descriptor: (Respiratory Tract Diseases) explode all tres) OR (“respiratory tract disease”):ti,ab,kw OR (“lung transplant”):ti,ab,kw OR (“lung transplantation”):ti,ab,kw OR (“respiratory tract diseases”):ti,ab,kw OR (“pulmonary disease”):ti,ab,kw OR (“lung disease”):ti,ab,kw OR (“respiratory disease”):ti,ab,kw OR (“respiration disorders”):ti,ab,kw OR (“asthma”):ti,ab,kw OR (“asthmatic”):ti,ab,kw OR (“bronchitis”):ti,ab,kw OR (“bronchiectasia”):ti,ab,kw OR (“bronchitic”):ti,ab,kw OR (“bronchiectases”):ti,ab,kw OR (“bronchiectasic”:ti,ab,kw OR (“chronic obstructive pulmonary disease”):ti,ab,kw OR (“COPD”):ti,ab,kw OR (“lung cancer”):ti,ab,kw OR (“sleep apnea”:ti,ab,kw OR (“covid-19”):ti,ab,kw OR “covid 19”:ti,ab,kw OR (“covid19”):ti,ab,kw OR (“SARS-CoV-2”):ti,ab,kw)
**INTERVENTION**	((MeSH descriptor: (Nordic Walking) explode all tres) OR (“nordic walking”):ti,ab,kw OR (“walking with poles”):ti,ab,kw OR

	("polestriding"):ti,ab,kw OR ("nordic pole walking"):ti,ab,kw OR ("pole walking"):ti,ab,kw)
Note: Keyword groups will be linked by AND	

7. **Search strategy for Clinicaltrials.gov**

**PARTICIPANTS**	("Respiratory Disease" OR "Pulmonary Disease" OR "Lung disease" OR "Lung cancer" OR "Lung transplatation" OR "covid 19" OR "COVID-19" OR "asthma" OR "Bronchitis" OR "Bronchiectases" OR "sleep apnea" OR "Chronic Obstructive Pulmonary Disease")
**INTERVENTION**	("Nordic Walking" OR "walking with poles" OR "Nordic pole walking" OR "pole walking" OR "pole striding" OR "polestriding")
Note: Keyword groups will be linked by AND	

8. **Search strategy for BVS (LILACS and IBECS)**

**PARTICIPANTS**	((mh:("respiratory tract diseases")) OR (mh:("lung transplantation")) OR ("lung transplant") OR ("pulmonary disease") OR ("respiratory disease") OR ("lung disease") OR ("respiration disorders") OR ("asthm*") OR ("bronchit*") OR ("bronchiectas*") OR ("lung cancer") OR ("sleep apnea") OR ("chronic obstructive pulmonary disease") OR ("copd") OR ("covid-19") OR ("covid 19") OR ("covid19") OR ("SARS-CoV-2"))
**INTERVENTION**	((mh:("Nordic walking")) OR ("Nordic walking") OR ("Nordic pole walking") OR ("pole walking") OR ("pole striding") OR ("polestriding") OR ("walking with poles"))
Note: Keyword groups will be linked by AND	

9. **Search strategy for SPORTDiscus**

**PARTICIPANTS**	((MM "Respiratory Tract Diseases") OR "Respiratory tract diseas*" OR "lung transplant*" OR "pulmonary diseas*" OR "lung diseas*" OR "respiratory diseas*" OR "respiration disorder*" OR "asthm*" OR "bronchit*" OR "bronchiectas*" OR "chronic obstructive pulmonary disease" OR "COPD" OR (MM "COVID-19+") OR "covid19" OR "covid 19" OR "covid-19" OR "SARS-CoV-2" OR "Lung cancer" OR "SARS-CoV-2")
**INTERVENTION**	((MM "Nordic Walking") OR "nordic walking" OR "walking with poles" OR "pole striding" OR "Polestriding" OR "Nordic Pole Walking" OR "pole walking")
Note: Keyword groups will be linked by AND	

Appendix B. Risk of bias tool summary

Author	D1	D2	D3	D4	D5	Overall
Breyer, 2010	+	-	-	-	!	-
Jastrzebski, 2013	-	-	-	-	!	-
Jastrzebski, 2015	-	!	+	-	!	-
Rinaldo, 2017	+	!	!	-	!	-
Berger, 2018	-	-	-	+	!	-
Ochman, 2018	-	-	-	-	-	-
Cunningham, 2019	+	!	!	-	+	-
Ruban, 2019	-	!	+	-	!	-
Rutkowska, 2019	+	-	+	-	+	-
Kuzina, 2020	-	-	-	-	-	-
Acar, 2023	!	!	+	-	!	-
Sivagnanam, 2023	+	!	+	-	!	-
Yogeshwaran, 2024	+	-	!	-	!	-

+ Low risk
! Some concerns
- High risk

D1	Randomisation process
D2	Deviations from the intended interventions
D3	Missing outcome data
D4	Measurement of the outcome
D5	Selection of the reported result

Appendix C. Heterogeneity and sensitivity analysis

Heterogeneity analysis

Table C.1. Heterogeneity analysis of the comparison between NW and Control Group on 6 minute

s walking test (6MWT)

Subgroup analysis (nº studies)	sample NW/CG	MD	95% CI	I^2
All studies (7)	107/104	4.41	(-88.06, 96.88)	94%
Control Group Type				
No Intervention (4)	65/52	63.96	(16.22, 111.69)	0%
Active Intervention (3)	52/52	-47.49	(-224.69, 129.72)	98%

CI: Confidence Interval; I^2: Heterogeneity Statistic; MD: Mean Difference; NW: Nordic Walking; CG: Control Group

Sensitivity analysis

Table C.2. Sensitivity analysis of the comparison between NW and Control Group on 6 minutes walking test (6MWT)

Study dropped out	sample NW/CG	MD	95% CI	I^2
None	107/104	4.41	(-88.06, 96.88)	94%
Berger 2018	87/74	-11.59	(-119.44, 96.26)	95%
Cunningham 2020	114/100	6.28	(-93.09, 105.64)	95%
Jastrezbski 2015	105/96	-3.46	(-108.05, 101.14)	95%
Rinaldo 2017	105/92	-6.51	(-115.76, 102.75)	95%
Rutkowska 2019	97/94	-1.45	(-107.77, 104.87)	95%
Sivagnanam 2023	97/84	30.29	(21.47, 39.10)	0%
Yogeshwaran 2024	97/84	0.92	(-136.86, 138.69)	94%

CI: Confidence Interval; I^2: Heterogeneity Statistic; MD: Mean Difference; NW: Nordic Walking; CG: Control Group

Study or Subgroup	Nordic Walking Mean	Nordic Walking SD	Nordic Walking Total	Other Interventions Mean	Other Interventions SD	Other Interventions Total	Weight	Mean Difference IV, Random, 95% CI	Year
Breyer 2010	540	159	30	442	133	30	1.4%	98.00 [23.82, 172.18]	2010
Jastrezbski 2015	563.9	64.6	12	509.4	134.3	8	0.8%	54.50 [-45.48, 154.48]	2015
Rinaldo 2017	549	48	12	480	121	12	1.4%	69.00 [-4.65, 142.65]	2017
Rutkowska 2019	531	103	20	490	124	10	1.0%	41.00 [-48.13, 130.13]	2019
Cunningham 2020	512.3	153	3	523.6	46	4	0.2%	-11.30 [-190.21, 167.61]	2020
Yogeshwaran 2024	351.5	13.09	20	323	15.93	20	95.2%	28.50 [19.46, 37.54]	2024
**Total (95% CI)**			**97**			**84**	**100.0%**	**30.29 [21.47, 39.10]**	

Heterogeneity: Tau² = 0.00; Chi² = 4.90, df = 5 (P = 0.43); I² = 0%
Test for overall effect: Z = 6.73 (P < 0.00001)

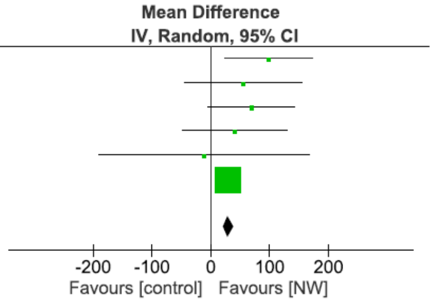

Appendix D. Meta-Analysis

Figure D.1. Comparison of 6 minutes walking test (6MWT) (m) between Nordic walking (NW) and control groups (CG) without Sivagnanam et al. study. CI: confidence interval; I²: heterogeneity statistic; IV: inverse variance

S1. PRISMA Checklist

Section and Topic	Item #	Checklist item	Location where item is reported
**TITLE**			
Title	1	Identify the report as a systematic review.	Line 4, 42, 44, 55, 107
**ABSTRACT**			
Abstract	2	See the PRISMA 2020 for Abstracts checklist.	Yes
**INTRODUCTION**			
Rationale	3	Describe the rationale for the review in the context of existing knowledge.	Lines 101-103
Objectives	4	Provide an explicit statement of the objective(s) or question(s) the review addresses.	Lines 102-104
**METHODS**			
Eligibility criteria	5	Specify the inclusion and exclusion criteria for the review and how studies were grouped for the syntheses.	Lines 111-122
Information sources	6	Specify all databases, registers, websites, organisations, reference lists and other sources searched or consulted to identify studies. Specify the date when each source was last searched or consulted.	Lines 123-140
Search strategy	7	Present the full search strategies for all databases, registers and websites, including any filters and limits used.	Appendix A
Selection process	8	Specify the methods used to decide whether a study met the inclusion criteria of the review, including how many reviewers screened each record and each report retrieved, whether they worked independently, and if applicable, details of automation tools used in the process.	Lines 141-147
Data collection process	9	Specify the methods used to collect data from reports, including how many reviewers collected data from each report, whether they worked independently, any processes for obtaining or confirming data from study investigators, and if applicable, details of automation tools used in the process.	Lines 148-152
Data items	10a	List and define all outcomes for which data were sought. Specify whether all results that were compatible with each outcome domain in each study were sought (e.g. for all measures, time points, analyses), and if not, the methods used to decide which results to collect.	Lines 116-120
	10b	List and define all other variables for which data were sought (e.g. participant and intervention characteristics, funding sources). Describe any assumptions made about any missing or unclear information.	Lines 112-116, 120-122
Study risk of bias assessment	11	Specify the methods used to assess risk of bias in the included studies, including details of the tool(s) used, how many reviewers assessed each study and whether they worked independently, and if applicable, details of automation tools used in the process.	Lines 154-169
Effect measures	12	Specify for each outcome the effect measure(s) (e.g. risk ratio, mean difference) used in the synthesis or presentation of results.	Lines 170-185
Synthesis methods	13a	Describe the processes used to decide which studies were eligible for each synthesis (e.g. tabulating the study intervention characteristics and comparing against the planned groups for each synthesis (item #5)).	Lines 171-173
	13b	Describe any methods required to prepare the data for presentation or synthesis, such as handling of missing summary statistics, or data	Lines 170-185

Section and Topic	Item #	Checklist item	Location where item is reported
		conversions.	
	13c	Describe any methods used to tabulate or visually display results of individual studies and syntheses.	Lines 170-185
	13d	Describe any methods used to synthesize results and provide a rationale for the choice(s). If meta-analysis was performed, describe the model(s), method(s) to identify the presence and extent of statistical heterogeneity, and software package(s) used.	Lines 170-185
	13e	Describe any methods used to explore possible causes of heterogeneity among study results (e.g. subgroup analysis, meta-regression).	Lines 181-182
	13f	Describe any sensitivity analyses conducted to assess robustness of the synthesized results.	Lines 182-183
Reporting bias assessment	14	Describe any methods used to assess risk of bias due to missing results in a synthesis (arising from reporting biases).	Lines 170-185
Certainty assessment	15	Describe any methods used to assess certainty (or confidence) in the body of evidence for an outcome.	Lines 170-185
**RESULTS**			
Study selection	16a	Describe the results of the search and selection process, from the number of records identified in the search to the number of studies included in the review, ideally using a flow diagram.	Lines 188-193 Figure 1.
	16b	Cite studies that might appear to meet the inclusion criteria, but which were excluded, and explain why they were excluded.	Figure 1.
Study characteristics	17	Cite each included study and present its characteristics.	Table 1. Lines 194-232
Risk of bias in studies	18	Present assessments of risk of bias for each included study.	Lines 233-240. Figure 2
Results of individual studies	19	For all outcomes, present, for each study: (a) summary statistics for each group (where appropriate) and (b) an effect estimate and its precision (e.g. confidence/credible interval), ideally using structured tables or plots.	Lines 241-315
Results of syntheses	20a	For each synthesis, briefly summarise the characteristics and risk of bias among contributing studies.	Lines 241-315
	20b	Present results of all statistical syntheses conducted. If meta-analysis was done, present for each the summary estimate and its precision (e.g. confidence/credible interval) and measures of statistical heterogeneity. If comparing groups, describe the direction of the effect.	Lines 243-260.Figure 3, Figure 4, Appendix C
	20c	Present results of all investigations of possible causes of heterogeneity among study results.	Lines 247-260 Appendix C
	20d	Present results of all sensitivity analyses conducted to assess the robustness of the synthesized results.	Lines 248-254 Appendix C
Reporting biases	21	Present assessments of risk of bias due to missing results (arising from reporting biases) for each synthesis assessed.	Figure 3, Figure 4, Appendix C,

Section and Topic	Item #	Checklist item	Location where item is reported
			Appendix D
Certainty of evidence	22	Present assessments of certainty (or confidence) in the body of evidence for each outcome assessed.	Figure 3, Figure 4, Appendix C, Appendix D
**DISCUSSION**			
Discussion	23a	Provide a general interpretation of the results in the context of other evidence.	Lines 317-407
	23b	Discuss any limitations of the evidence included in the review.	Lines 409-436
	23c	Discuss any limitations of the review processes used.	Lines 409-436
	23d	Discuss implications of the results for practice, policy, and future research.	Lines 360-254, 407, 449-450
**OTHER INFORMATION**			
Registration and protocol	24a	Provide registration information for the review, including register name and registration number, or state that the review was not registered.	Lines 107-110
	24b	Indicate where the review protocol can be accessed, or state that a protocol was not prepared.	Lines 107-110
	24c	Describe and explain any amendments to information provided at registration or in the protocol.	NA
Support	25	Describe sources of financial or non-financial support for the review, and the role of the funders or sponsors in the review.	Lines 454
Competing interests	26	Declare any competing interests of review authors.	Line 453
Availability of data, code and other materials	27	Report which of the following are publicly available and where they can be found: template data collection forms; data extracted from included studies; data used for all analyses; analytic code; any other materials used in the review.	Lines 457-458

From: Page MJ, McKenzie JE, Bossuyt PM, Boutron I, Hoffmann TC, Mulrow CD, et al. The PRISMA 2020 statement: an updated guideline for reporting systematic reviews. BMJ 2021;372:n71. doi: 10.1136/bmj.n71
